# Benchmarking Bioinformatic Tools for Amplicon-Based Sequencing of Norovirus

**DOI:** 10.1128/aem.01522-22

**Published:** 2022-12-21

**Authors:** Amy H. Fitzpatrick, Agnieszka Rupnik, Helen O’Shea, Fiona Crispie, Sinéad Keaveney, Paul D. Cotter

**Affiliations:** a Department of Food Biosciences, Teagasc Food Research Centre, Fermoy, Ireland; b Department of Shellfish Microbiology, Marine Institute, Oranmore, Ireland; c Department of Biological Sciences, Munster Technological University, Bishopstown, Ireland; Centers for Disease Control and Prevention

**Keywords:** high-throughput sequencing, *in silico*, classification, denoising, clustering, environmental virology, *Caliciviridae*, calicivirus

## Abstract

In order to survey noroviruses in our environment, it is essential that both wet-lab and computational methods are fit for purpose. Using a simulated sequencing data set, denoising-based (DADA2, Deblur and USEARCH-UNOISE3) and clustering-based pipelines (VSEARCH and FROGS) were compared with respect to their ability to represent composition and sequence information. Open source classifiers (Ribosomal Database Project [RDP], BLASTn, IDTAXA, QIIME2 naive Bayes, and SINTAX) were trained using three different databases: a custom database, the NoroNet database, and the Human calicivirus database. Each classifier and database combination was compared from the perspective of their classification accuracy. VSEARCH provides a robust option for analyzing viral amplicons based on composition analysis; however, all pipelines could return OTUs with high similarity to the expected sequences. Importantly, pipeline choice could lead to more false positives (DADA2) or underclassification (FROGS), a key aspect when considering pipeline application for source attribution. Classification was more strongly impacted by the classifier than the database, although disagreement increased with norovirus GII.4 capsid variant designation. We recommend the use of the RDP classifier in conjunction with VSEARCH; however, maintenance of the underlying database is essential for optimal use.

**IMPORTANCE** In benchmarking bioinformatic pipelines for analyzing high-throughput sequencing (HTS) data sets, we provide method standardization for bioinformatics broadly and specifically for norovirus in situations for which no officially endorsed methods exist at present. This study provides recommendations for the appropriate analysis and classification of norovirus amplicon HTS data and will be widely applicable during outbreak investigations.

## INTRODUCTION

Human noroviruses (NoV) are the leading cause of acute gastroenteritis in humans (1). The 10 known NoV genogroups that have been recognized (GI to GX) can be further subdivided into at least 49 different genotypes (2, 3). Norovirus is transmitted via the fecal-oral route, most often person-to-person or via contaminated foodstuffs or water (4). Foodborne transmission can occur either when food handlers contaminate food or when shellfish are grown in harvesting areas subject to wastewater overflow (4). There have been several documented outbreaks of norovirus related to shellfish and berry consumption (5–7).

Currently, quantitative reverse transcription PCR (RT-qPCR) methods are widely applied for the surveillance of noroviruses in shellfish and, more recently, for leafy greens and berries (8). The ORF1-ORF2 junction is the most conserved region of the genome and therefore the site of targeted amplification as per ISO 15216-1:2017 (9). The ISO method allows for relative quantification based on a standard curve, generated from double-stranded DNA (dsDNA) (in annex G of ISO 15216-1:2017), and can discriminate between genogroups. The method includes a suite of controls to address issues such as contamination of reagents, cross-contamination, and inhibition (8). However, in the case of foodborne outbreaks, where source attribution is required, genogroup level classification is often not sufficient to link the suspected foodstuff and clinical cases. Food may be contaminated with more than one genogroup of norovirus at a time. Furthermore, the current oversampling of clinical cases in institutional settings gives a poor understanding of norovirus emergence and persistence (10). It is important to develop high-throughput sequencing (HTS)-based methods capable of discriminating noroviruses at a genotypic level in food and environmental samples to address this gap in the data and to permit source attribution in foodborne outbreaks.

Wet-lab methods for food and environmental samples will likely rely on amplicon-based sequencing due to the need to overcome challenges associated with (i) low levels of viral contamination, (ii) poor quality degraded RNA due to exposure to UV light and wastewater treatment processes such as chlorination, and (iii) challenges of working with specific matrices, such as inhibition caused by humic acids in shellfish and polyphenols in berries. As currently implemented, shotgun metagenomics performed without modifications, such as capture probe hybridization or rRNA removal, are unlikely to yield high-quality norovirus genomes in such samples (11–13).

Following the generation of sequence data, bioinformatic tools are of key importance for accurately typing norovirus. It is not certain that the currently applied 16S/18S/ITS rRNA bacteria/fungal clustering and denoising approaches are suitable for the analysis of viral amplicons, due to (i) the lack of conserved marker genes in viruses (14), (ii) high genetic diversity (2, 10, 15), and (iii) the existence of quasispecies (6, 16, 17). The field has primarily focused on the wet-lab methodology (12, 13, 18). Optimizing the bioinformatic pipelines employed is essential if one wants to apply HTS data for analysis of clinical or regulatory significance, such as source attribution in foodborne outbreaks.

Clustering and denoising methods have been established to both reduce redundancy and overcome sequencing errors. Clustering-based methods try to combine a set of sequences into meaningful biological entities called operational taxonomic units (OTUs) (19–21). Usually, only one representative sequence from each OTU is kept, but this is not true across all clustering pipelines. Denoising exploits the observation that a low abundance sequence that is very similar to a high-abundance sequence is likely to be an error. The central challenge of denoising is determining an abundance threshold that discriminates a correct sequence from an error. Error frequencies vary due to biases that cannot be accurately predicted and to fluctuations due to sampling effects that are predictably present but have unpredictable values for any given sequence. It is also important to note that some current denoising methods (DADA2, Deblur, and USEARCH-UNOISE3) can be applied only for Illumina-generated sequencing outputs and, in some cases, PacBio, as the error profiles are specific to the platform.

The objective of this study was to optimize and benchmark a bioinformatic pipeline and classifier for rapid investigation of foodborne outbreaks of norovirus. The complexity of foodborne outbreaks, from the matrix, sampling time and comparison to clinical ‘ground truth’ sequences, requires a high degree of standardization and accuracy across jurisdictions (22, 23). This is the first evaluation of pipelines and classifiers for bioinformatic analysis of norovirus HTS amplicons.

## RESULTS

Multiplexing in environmental virology is important for both cost and study-design implications; therefore, a dual index strategy was employed here. As the objective is to provide a bioinformatic method that permits the accurate genotypic characterization of norovirus in environmental samples, multiple contamination events are considered. As shellfish are contaminated by wastewater over an undefined period, a mixture of genotypes is expected. Typically, contamination presents with one dominant population, which reflects the decay rate of norovirus throughout bioaccumulation and the expected log-normal distribution of the virus (24). Therefore, 2 to 10 genotypes were assigned per sample (7).

### Pipeline choice significantly impacted compositional output with UNOISE3 and VSEARCH performing best.

**FIG 1:**
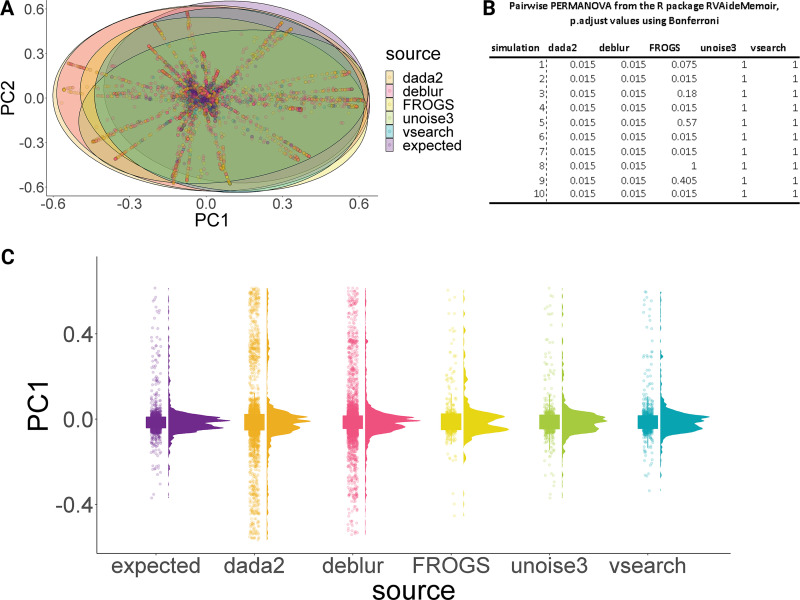
(A) Bray-Curtis dissimilarity matrix demonstrates the overlap between expected and observed relative abundance of amplicon sequencing variants (ASVs)/operational taxonomic units (OTUs)/zero-radius OTUs (zOTUs) generated using the selected approaches. (B) Differences across Bray-Curtis dissimilarity across simulations were assessed using a type II permutation multivariate analysis of variance (MANOVA) that returned an *R*^2^ value of 0.009 to 0.015, indicating that the pipeline did not contribute to the variation in distances (*P* = 0.0001). A pairwise nonparametric multivariate statistical permutation test (PERMANOVA) indicated a significant difference between expected composition and obtained composition from DADA2 and Deblur, adjusted *P* = 0.015 (Bonferroni), whereas USEARCH-UNOISE3 and VSEARCH pipelines yielded an adjusted *P* value of 1, indicating high similarity between obtained and observed composition. (C) Raincloud and box plots of the Bray-Curtis dissimilarity matrix based on the primary principle component demonstrates that DADA2 output was significantly different from the expected composition, followed by Deblur and FROGS.

Analysis of similarities (ANOSIM, nonparametric test) was used to identify significant differences between outputs from pipelines based on Bray-Curtis distance output from the *R* package vegan, vegdist (25). This returned an *R*^2^ value of −0.003 to 0.01 with a *P* value of 0.83 to 1, indicating high similarity between compositional data sets, with greater within-group variation than between-group variation. A type II permutation multivariate analysis of variance (MANOVA) using distance matrices was performed on the Bray-Curtis distance matrix, as seen in Fig. 1A, and an *R*^2^ value of 0.009 to 0.015 was obtained, indicating that the pipeline did not contribute to the variation in distances (*P* = 0.0001). A *post hoc* test on the Bray-Curtis distance matrix was performed using pairwise nonparametric multivariate statistical permutation test (PERMANOVA) from the *R* package RVAideMemoir (26) with 999 permutations, which indicated a significant difference between the expected composition and the obtained composition from DADA2 and Deblur, *P* value adjusted 0.015 (Bonferroni corrected), whereas USEARCH-UNOISE3 and VSEARCH pipelines yielded an adjusted *P* value of 1, indicating high similarity between the expected and observed compositions (Fig. 1B). A Wilcoxon test was used to discriminate whether there significant compositional differences between the expected and observed data from pipeline/simulation (Fig. S1). As evident, DADA2, Deblur, FROGS, USSEARCH-UNOISE3, and VSEARCH were significantly different 4/10, 1/10, 3/10, 0/10, and 0/10 times. In conclusion, pipeline choice significantly impacted the accurate representation of compositional data. VSEARCH returned the most similar compositional data set, while DADA2 and Deblur returned the least similar data set, as per pairwise PERMANOVA (Fig. 1B).

### Differences in pipeline performance were not due to low abundance OTUs nor specific genotypes.

**FIG 2:**
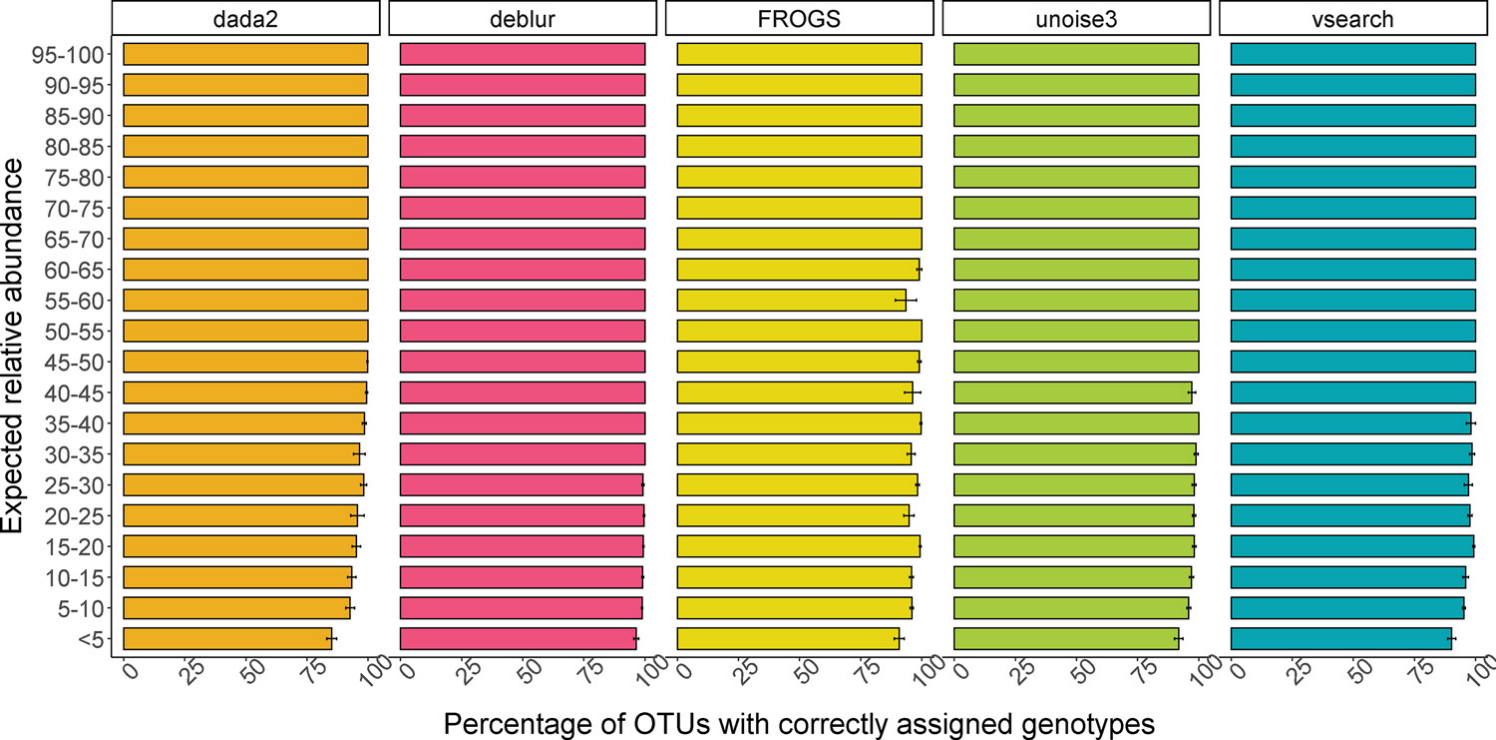
Box plot demonstrating the percentage of correctly assigned genotypes at each abundance level (see *y* axis). Error bars represent the standard error of the mean percentage of correctly assigned genotypes across each simulation.

A Kruskal-Wallis test from the rstatix package in *R* was used to determine whether low abundance OTUs were less likely to be classified at genotype level than higher abundance OTUs. All pipelines demonstrated varied performance across expected relative abundance, as shown in Fig. 2 (*P* < 0.05; see Table S1 in supplemental material). Specifically, OTUs present at <5%, 5 to 10%, 10 to 15%, and 30 to 35% were significantly less likely to be correctly classified at genotype level (*P* < 0.05, Kruskal-Wallis). A Dunn test (rstatix) with Bonferroni correction was used to assess whether specific pipelines were more accurate at typing lower abundant OTUs. No significant differences were observed, regardless of the expected relative abundance level (low to high).

**FIG 3:**
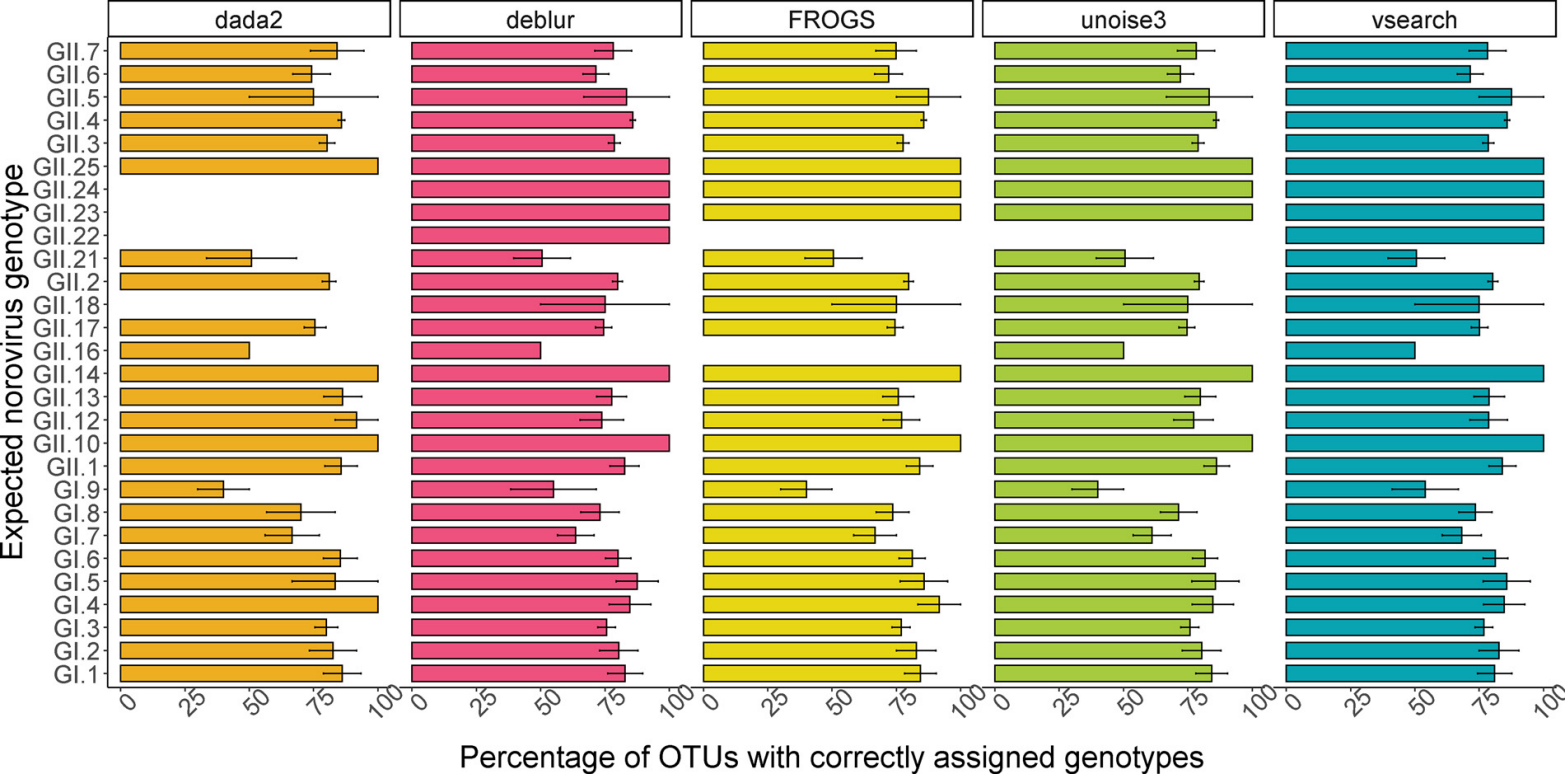
Box plot demonstrating the percentage of correctly assigned genotypes at each expected norovirus genotype (see *y* axis). Error bars represent the standard error of the mean percentage of correctly assigned genotypes across each simulation.

There were no significant differences in performance by pipeline when accounting for genotype. As some genotypes were present in only one sample in one simulation, there was not sufficient statistical power to determine pipeline performance in relation to these missed genotypes, for example GII.24 and GII.18. However, as can be seen in Fig. 3, there is a clear difference in performance for specific genotypes, regardless of pipeline. VSEARCH and Deblur were able to generate OTUs/amplicon sequencing variants (ASVs) for all genotypes included in this study.

### Pipeline choice did not impact phylogenetic distance from the expected sequences.

**FIG 4:**
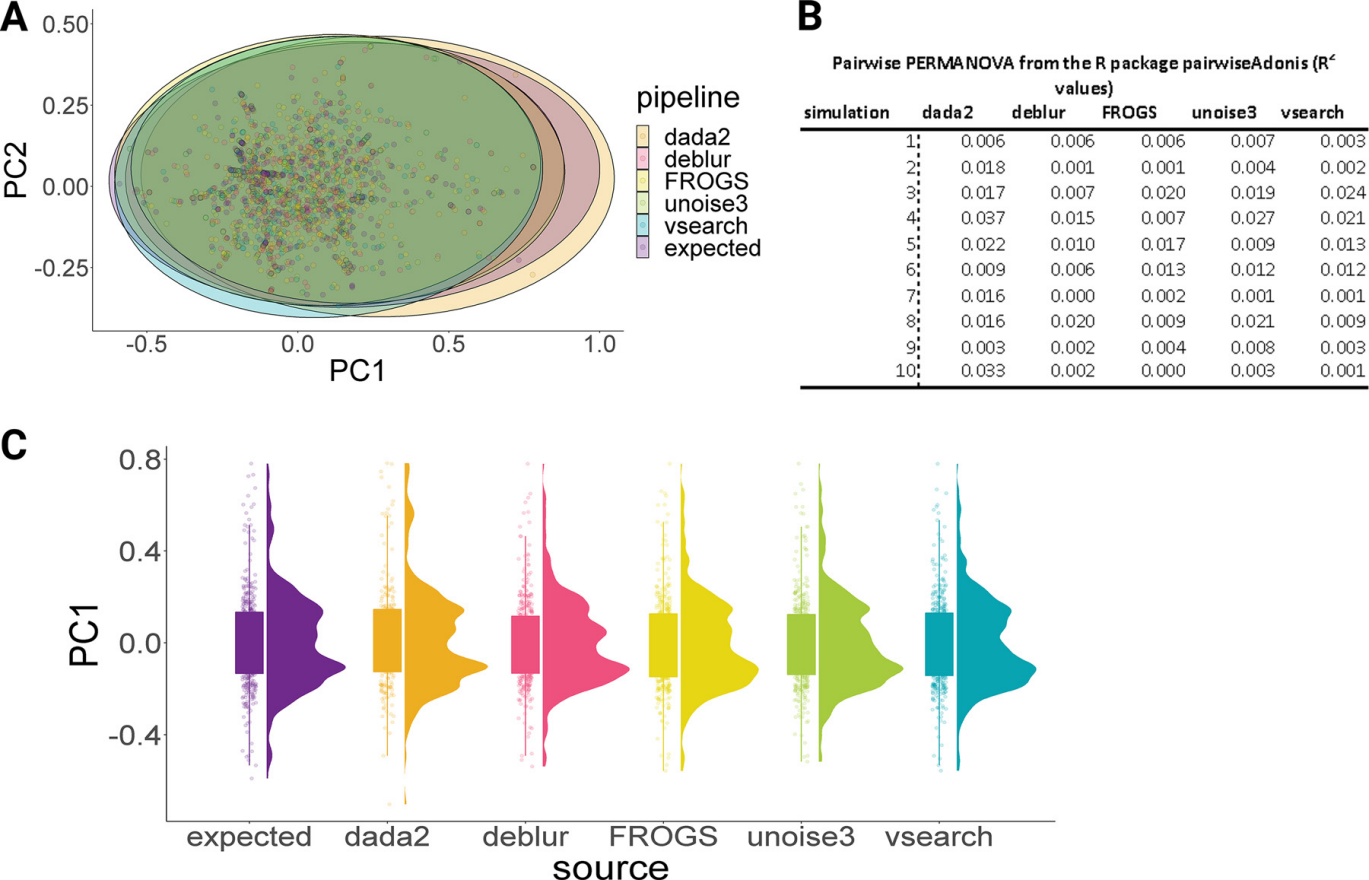
(A) PCOA of weighted UniFrac (QIIME2) across all simulations reveals overlap between expected and observed sequences (*R*^2^ = −0.08, *P* = 1). (B) PERMANOVA *R*^2^ values center around 0, with no pipeline/simulation providing a *R*^2^ value of greater than 0.037. (C) Raincloud and box plots of weighted UniFrac based on the primary principal component. There were no significant differences between expected sequences and observed sequences using the Wilcoxon test, and overall, no differences between sequences obtained using any of the pipelines (Kruskal-Wallis).

A Wilcoxon test was used to evaluate whether UniFrac distances, unweighted and weighted (Fig. S2 and S3), were significantly different between the expected and observed sequences. For both weighted and unweighted UniFrac, no significant differences were observed between the expected and observed sequences, even with abundance accounted for (weighted UniFrac Fig. 4C). There were no differences between the pipelines (Kruskal-Wallis test) (Fig. S3) for results for each simulation. Across all simulations, weighted UniFrac demonstrated a high degree of similarity between sequences obtained from all pipelines (Fig. 4B). Furthermore, ANOSIM returned a low *R*^2^ value of −0.08 and a *P* value of 1, highlighting that the pipeline did not contribute to any differences between values. A pairwise PERMANOVA from the *R* package RVAideMemoir (26) was used to investigate the *R*^2^ value across the pipeline and simulation combinations, and all *R*^2^ values centered on 0 (Fig. 4B). This underlines once again that the pipeline did not affect phylogenetic distances between the expected and observed sequences. In conclusion, while pipeline choice affected compositional accuracy, it had little impact on the accuracy of amplicon sequences derived from the simulated sequencing data.

**FIG 5:**
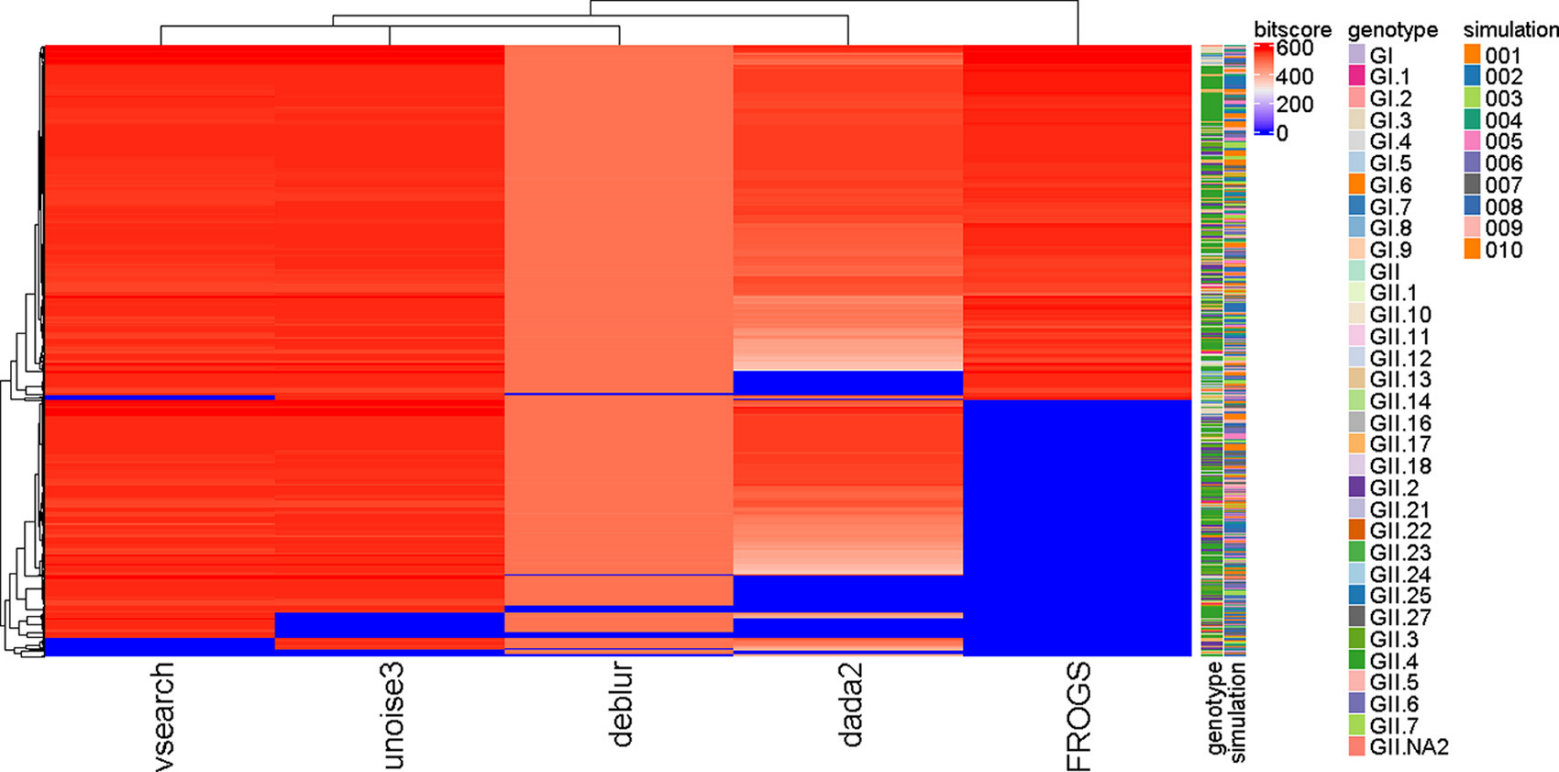
Heat map of bit scores returned when observed sequences were subject to blastn at 99% identity with 75% coverage. There were shared patterns of lower bit scores for specific sequences across all five pipelines. VSEARCH and UNOISE3 appeared to have improved performance compared to other pipelines. ANOSIM returned a *P* value of 0.001 but an *R*^2^ value of 0.02, indicating that the pipeline choice had a small impact on sequence similarity. PERMANOVA revealed that FROGS had significantly different bit scores to all pipelines (*P*-value < 0.05).

Observed sequence similarity was acceptable (>50) in all pipelines. UNOISE3 and VSEARCH outperformed other pipelines, but this was not found to be statistically significant. To differentiate between the performance of pipelines in terms of sequence identity and quality of those sequences, pipelines were compared based on bit score (Fig. 5) and *E* value (Fig. S4).

ANOSIM was used to investigate differences based on the bit score and *E* value obtained across pipelines. For bit score, ANOSIM returned a *P* of 0.001 but an *R*^2^ value of 0.02, indicating that the pipeline choice had a small impact on sequence similarity. PERMANOVA (26) revealed that FROGS had bit scores significantly different from all other pipelines (*P* < 0.05). ANOSIM for the *E* value returned an *R*^2^ value of 0.1 and a *P* value of 0.001, indicating that pipeline choice had a minimal impact on *E* value. A *post hoc* test on the Bray-Curtis distance matrix of E values was performed using pairwise PERMANOVA (26). There were patterns in the OTUs that returned lower bit scores; as can be seen by the dark blue lines shared across all pipelines for specific OTUs (Fig. 5). Red indicates very high bit scores of greater than 400, while dark blue indicates a bit score >50 but <100. The bit score is linearly related to the raw alignment score. Thus, the higher the bit score, the more highly significant the match is.

Subsequently, the quality of these sequences varied. While all returned acceptable bit scores (>50) and E values (<0.01), as clearly demarcated in Fig. 5, USEARCH-UNOISE3 and VSEARCH consistently returned high-quality sequences (bit score).

In terms of expected sequences for which 99% matches could not be obtained, a Kruskal-Wallis test was performed to determine whether there were significant differences in terms of the genotypes missed by each pipeline. While there was no significant difference between pipelines (*P* = 0.91), the most common genotypes that did not return a 99% BLASTn match included GII.4_Den_Haag, GII.2, GII.17, GII.3, GI.3, GII.4, GI.6, GII.4_Farmington_Hills, GII.4_Sydney, and GII.6. A Kruskal-Wallis test was used to determine whether specific genotypes were more likely to be missed, but again, there were no significant differences observed (*P* = 0.85 to 0.11).

### RDP classifier performed best with the custom or NoroNet database.

**FIG 6:**
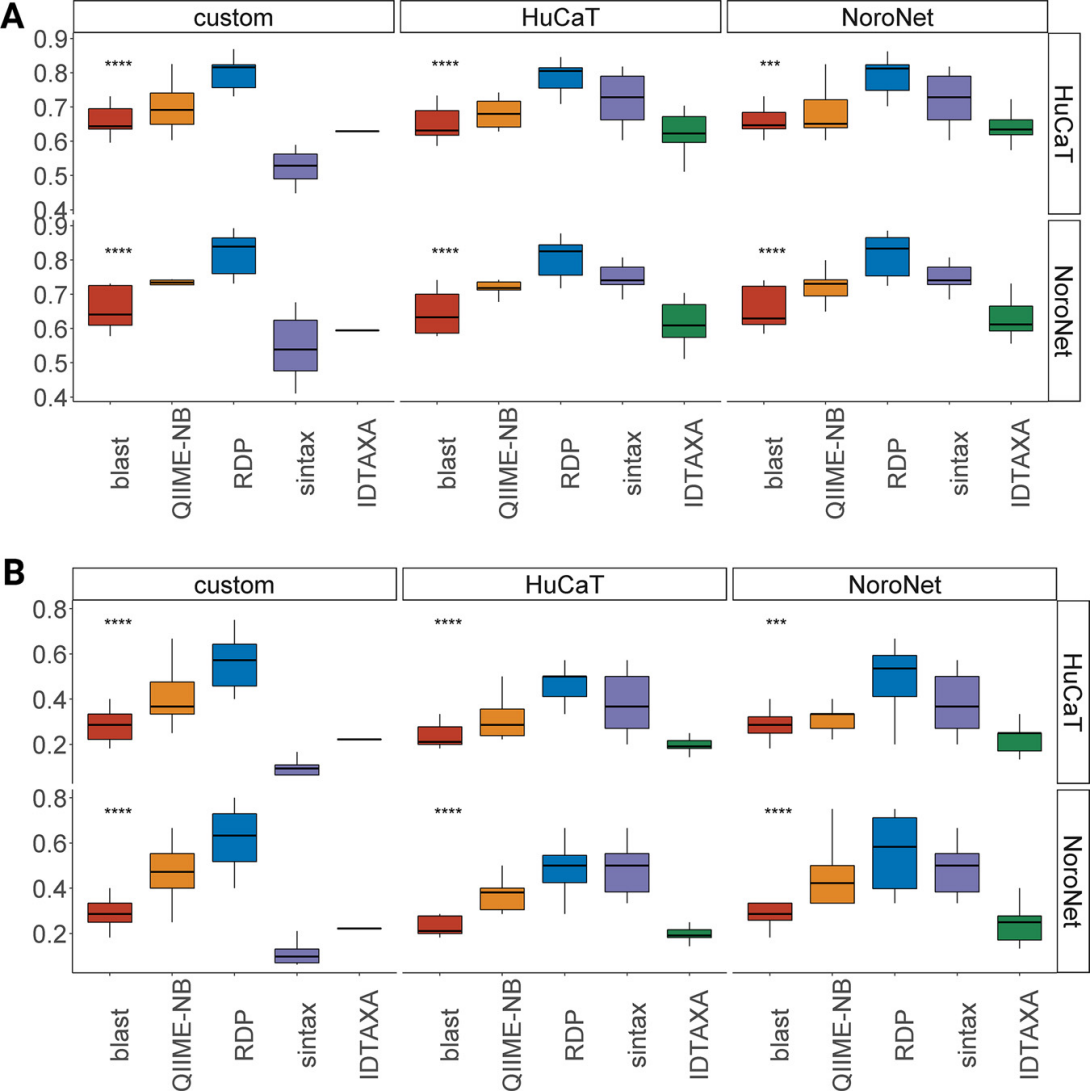
(A) Box plot of balanced accuracy at taxonomic assignment to genotypic level. Classifiers were significantly different from one another (Kruskal-Wallis test) (B) Box plot of F1 harmonic score at taxonomic assignment to genotypic level. Classifiers were significantly different from one another (Kruskal-Wallis test, (3 × 10^−8^ to 1.2 × 10^−4^).

Using the confusion matrix in the *R* package yardstick (27), classifiers and databases were compared based on the sensitivity or true positive rate (TPR), false-positive rate (FPR) or 1-specificity, F1 score, and balanced accuracy (average of sensitivity and specificity). The five classifiers chosen for comparison were IDTAXA, BLASTn, RDP, QIIME2-naive Bayes, and SINTAX. The Kruskal-Wallis test was used to assess TPR, F1 score, and balanced accuracy and indicated significant differences across all classifiers. The FPR was not significantly different across classifiers run with the NoroNet database compared to the NoroNet typing tool (Fig. S6). In terms of differences between individual classifiers, BLASTn was significantly different (Dunn *post hoc* test rstatix) from other classifiers, in particular RDP, based on F1 score and balanced accuracy (Tables S2 and S3; Fig. 6). As noted in Fig. 6 (A and B), classification was to the genotype level, not including capsid variant for norovirus GII.4 genotypes. Inclusion of capsid variants resulted in an increase in the FPR and a small drop in overall balanced accuracy and F1 score (Fig. S7, A to C). RDP performed well across balanced accuracy and F1 score metrics (Fig. 6A and B).

### Classifier affected the accuracy of taxonomic assignment more than the database.

To elucidate which factor had a stronger impact on classification accuracy (TPR, FPR, balanced accuracy, and F1 score), the scores were compared by performing a MANOVA with ADONIS on the Bray-Curtis distance metric (Table 1). Based on the high F statistic and reasonable *R*^2^ values returned for the relationship between the classifiers and accuracy at genotypic taxonomic designation, classifiers had a stronger impact on classification accuracy than the database. This is evident in Fig. 6, which displays a clear pattern of performance for all classifiers, regardless of the database, aside from SINTAX in combination with the custom database.

**TABLE 1:** MANOVA with ADONIS on the Bray-Curtis distance metric of a confusion matrix for classifier performance^*a*^

Factor	Taxonomic level	Standard	*R* ^2^	*F*	*P*_r_(>*F*)
Classifier	Genotype	NoroNet typing tool	0.425	79.058	0.001
Classifier	Genotype	NoroNet typing tool	0.402	34.124	0.001
Classifier	Genotype with capsid	Human calicivirus typing tool	0.455	33.626	0.001
Classifier	Genotype with capsid	Human calicivirus typing tool	0.382	24.938	0.001
Database	Genotype	NoroNet typing tool	0.029	10.881	0.001
Database	Genotype	NoroNet typing tool	0.026	4.454	0.005
Database	Genotype with capsid	Human calicivirus typing tool	0.022	2.813	0.042
Database	Genotype with capsid	Human calicivirus typing tool	0.011	1.610	0.174

a ADONIS, analysis of variance using distance matrices; MANOVA, multivariate analysis of variance.

## DISCUSSION

Environmental virology has experienced a renaissance due to the COVID-19 pandemic and renewed interest in wastewater surveillance, building on previous applications for enteric viruses (28–31). However, there remains room for improvement with respect to the tools and standardized methodologies that are available to manage viral foodborne outbreaks. While much work has been conducted to date to understand the limits of shotgun metagenomics and amplicon-based sequencing for genotypic characterization of norovirus in foodstuffs (32, 33), there has been only one bioinformatic study to date (18). Importantly, this single study did not focus on amplicon-based approaches or foodstuffs. A recent scientific report from the European Food Safety Authority applied FROGS for the assessment of norovirus OTUs, alongside additional measures for chimera identification and removal (12). However, no bioinformatic pipeline comparison was included in their method development.

Theoretically, the application of denoising-based methods to resolve sequences to one-nucleotide differences should benefit viral amplicon data sets due to their high diversity. However, based on the Bray-Curtis distance matrix, assessing dissimilarity in observed composition, USEARCH-UNOISE3 (denoising) and VSEARCH (clustering) best reflected the expected composition. Approaches such as swarm (FROGS) and VSEARCH rely on clustering amplicon sequences into operational taxonomic units (OTUs), based on an arbitrary sequence identity threshold (20, 21). Typically for bacteria, this threshold has been set at 97%. The disadvantage of this approach is that resolution can be lost as sequences below the identity threshold cannot be differentiated and are lost to downstream analysis. In the case of viral quasispecies or highly diverse viral amplicons, a one-nucleotide difference could represent a true biological OTU. In this study, the threshold was set to 99% in VSEARCH to capture viral diversity across genotypes (16, 34). UNOISE3 sets the clustering threshold at 100% and identifies errors based on abundance differences; producing a zero-radius operational taxonomic unit (zOTU) (35). High identity thresholds are important in accurately reflecting the expected data.

DADA2 and Deblur underrepresented the expected composition and are both denoising-based pipelines that have performed well in previous comparative studies focusing on 16S/18S/ITS data (34, 36–38). Deblur operates on each sample independently, comparing sequence-to-sequence Hamming distances within a sample to an upper-bound error profile and outputting suboperational taxonomic units (sOTUs) (36). In DADA2, sample composition is inferred by dividing amplicon reads into partitions consistent with the error mode, outputting amplicon sequencing variants (ASVs) (34). As evident in the results section (Fig. 1), DADA2 and Deblur generated spurious sOTUs/ASVs. It appears that the denoising algorithm employed in UNOISE3 is advantageous over DADA2/Deblur for the genotypic characterization of norovirus HTS amplicons. Importantly, low abundance OTUs were not driving differences in pipeline performance.

If compositional results vary, we must question what weight should be given to the relative abundance of genotype X over genotype Y. In the case of norovirus contamination of foodstuffs, conventional PCR or RT-PCR assays rely on a high number of PCR cycles to generate sufficient quantities of DNA for downstream sequencing. Moreover, traditionally, the VP1 of norovirus has been targeted using semi-nested PCR methods. It has been confirmed in numerous studies that PCR cycling number and polymerase fidelity can affect the quality of DNA obtained (39–43). In addition to this, primer-bias can result in preferential amplification of certain genotypes, skewing the relative abundance observed following bioinformatic analysis (44–46). Considering the applicability of this pipeline in a food outbreak setting, attempting to link food, environmental, and patient sequences, then phylogenetic measurements such as UniFrac rank higher than rank-based methods such as Bray-Curtis.

UniFrac distance measures the phylogenetic distance between sets of taxa in a phylogenetic tree as the fraction of the branch length of the tree that leads to descendants from either one environment or the other (47). UniFrac distance methods revealed that the pipeline had little impact on phylogenetic distances. Pivotally, BLASTn comparisons demonstrated that all pipelines could return sequences with a 99% identity, alongside acceptable bit scores and *E* values. However, VSEARCH and USEARCH-UNOISE3 returned sequences with higher bit scores and lower *E* values than other pipelines. If discrimination is to be made in terms of performance, there is a strong case for VSEARCH and USEARCH-UNOISE3 based on both the Bray-Curtis and BLASTn results.

The classifiers compared rely on a variety of methods to assign taxonomy (k-mer, BLASTn, naive Bayes, and decision tree). RDP is a naive Bayes classifier that assigns taxonomy based on repeated random sampling of 8-length k-mers and calculates confidence as the fraction of random samples that were assigned to a given taxonomic label (48). RDP had the best performance metrics regardless of the underlying database across all measures of classification accuracy. SINTAX’s performance was influenced by the underlying database, with lower classification accuracy when applied with the custom database. SINTAX identifies the taxonomy in each iteration by the top k-mer hit and sets the k-mer at 32 (49). While it performed well with the HuCaT and NoroNet databases, it exhibited greater variation in performance across simulation than the RDP and exhibited poor performance with the custom database. The key to performance variation across classifiers RDP, Naïve-Bayes QIIME2, and SINTAX may be due to the specified k-mer length (8,6 and 32). Future work should address k-mer length in norovirus classifier optimization (50, 51). IDTAXA and SINTAX have been designed to permit classification of novel species, and previous studies have demonstrated IDTAXA’s sensitivity to parametrization (52) and SINTAX’s poor performance (53). The relatively high confidence thresholds in BLASTn at the family level classification (90%) likely reduced the number of OTUs classified at genogroup and genotype taxonomic level. Furthermore, local pairwise alignments such as BLAST cannot accurately distinguish between highly similar sequences, nor can they cope with multifurcation or within-taxon variability, common among RNA viruses (54). Accordingly, we recommend the use of the RDP classifier alongside the gold-standard databases for the genotypic classification of norovirus within a bioinformatics pipeline.

There are limitations to this study. First and foremost, the sequencing data were simulated. It was not possible to retrieve norovirus sequencing read archive files that were from studies with (i) foodstuffs, (ii) amplicons, or (iii) Illumina platforms. Ideally, this work should be further validated with spiked foodstuffs of known concentrations and genotypes. It would be of particular interest to see the impact of PCR or seminested PCR on expected versus observed relative abundance.

Likewise, this study is limited to the VP1 region of norovirus or the capsid, and we have not demonstrated results for the RdRp or other Caliciviridae. Repeating this work with the RdRp may result in different outcomes due to the presence of the recombination breakpoint, increasing diversity across the amplicon (54, 55). This presents many challenges from a molecular detection and bioinformatics processing perspective. First, amplicons targeting the RdRp are short, and multiplex approaches are required to capture the diversity of the 60 known polymerase types (P-types). Furthermore, if multiple variable length fragments are sequenced on an Illumina platform, the shorter amplicons will be preferentially sequenced. Appropriate typing of P-types requires the identification of RdRp RT-PCR assay(s) that can capture the diversity of norovirus GI and GII P-types with satisfactory sensitivity and specificity. In terms of adjusting the pipelines, as the RdRp amplicons are typically shorter, minimum and maximum lengths and minimum overlaps should be adjusted, theoretically to lower values. Clustering identity for the VP1 capsid region has been set to 99% nucleotide identity, but this would need to be fine-tuned for polymerase types by comparing various clustering levels on a database of known P-types to P-type assignment in a validated classifier such as NoroNet or HuCat.

Second, if the objective is to study virus recombination, an amplicon spanning the RdRp-VP1 is required in environmental samples, as it is highly challenging to assemble full-length RdRp-VP1 from amplicons (56). The shortest published PCR assay targeting the RdRp-VP1 is 570 bp, and therefore an Oxford Nanopore Technology (ONT)/PacBio platform is required for sequencing, owing to read length limitations on Illumina platforms (57).

Finally, there is a degree of uncertainty in the ability of chimera detection tools to distinguish between PCR-mediated recombinants; also known as chimeras and historical homologous recombination events (58). Chimera removal should not remove homologous recombination events if they occur frequently within the viral population, as chimera removal accounts for relative abundance and/or targets template switching rather than incorporation of within strand fragments. Recombination detection from RdRp-VP1 amplicons spanning the breakpoint would permit detection of recombination events with a high degree of confidence in composite samples such as wastewater, but new/rare recombination events in clinical samples such as from immunocompromised patients may be difficult to detect. The accuracy of chimeras detected in HTS studies should be addressed in future work by performing an *in silico* PCR and HTS run with Simera (59) and InSilicoSeq, respectively, focusing on the ability of chimera detection tools to detect true PCR-mediated recombination events and subsequent homologous recombination detection through RDP (60).

For sequencing studies targeting the RdRp-VP1 fragment, the higher error profile of ONT platforms will have two subsequent impacts on the pipelines presented here. The first being that denoising pipelines such as DADA2, Deblur, and UNOISE3 use platform-specific error correction and cannot be used for ONT sequencing data currently. FROGS and VSEARCH can be applied to ONT sequencing output, but the higher error rate requires adjusting the clustering identity to account for single-nucleotide polymorphisms (SNPs) introduced by basecalling errors. Alternatively, Medaka may be more appropriate because it relies on neural networks to generate consensus sequences (61). Finally, other pipelines, classifiers and platforms were available for comparison, but the study was limited to those that have been widely applied and compared in previous studies focusing on 16S/18S/ITS.

In this study, we have demonstrated that pipeline choice affects compositional results but has a negligible impact on the similarity of sequences obtained. Furthermore, classifier choice accounted for accuracy more than the underlying database. Based on this study, we recommend that bioinformatic analysis of norovirus HTS amplicons be processed using VSEARCH and an RDP classifier in combination with a gold-standard database. For taxonomic classification of GII.4 capsid variants, gold standard typing tools should be used.

## MATERIALS AND METHODS

To evaluate pipelines, 10 MiSeq v3 sequencing runs were simulated with an average error rate. Clustering-based pipelines FROGS and VSEARCH and denoising-based pipelines DADA2, Deblur, and USEARCH-UNOISE3 were applied to simulated data. FROGS uses swarm to perform clustering (20). Swarm is an aggregative and unsupervised clustering method that utilizes a single linking clustering algorithm. Pipelines were compared to the known composition and sequences in the simulated data sets using a variety of measures. Two gold-standard typing tools for norovirus are available, one from RIVM, i.e., the NoroNet typing tool, and one from the Centers for Disease Control and Prevention (CDC), i.e., the human calicivirus typing tool (HuCaT) (62, 63). However, both tools are available for use only through online servers, creating a break in the processing of sequencing data. Furthermore, the NoroNet typing tool can take a significant amount of time to type large data sets; therefore, other methods of classification may have a time-result advantage in outbreak situations. The RDP classifier, QIIME2 naive Bayes, BLASTn, SINTAX, and IDTAXA classifiers were applied using the (i) NoroNet, (ii) HuCaT, or (iii) a custom database and compared on their classification accuracy.

### Pipeline comparison.

**(i) Data retrieval and standardization.** All available norovirus sequences consisting of between 2,000 and 7,500 bp (*n* = 4,420) were downloaded from GenBank using the *R* package rentrez (64). Reference sequences used in NoroNet and HuCaT typing tools were excluded as they could perform better than expected, biasing results if included. Classifiers are trained using specific sequence databases and when challenged with their training data set would be expected to perform well. Randomly picked sequences are more likely to challenge the classifiers. An additional 10,000 caliciviridae non-norovirus sequences were downloaded from GenBank (2,000 to 8,000 bp) in order to test primer specificity during the amplicon generation step. The output fasta files were combined, ambiguous bases were masked using EMBOSS (version 6.6.0), and duplicates were removed using bbmap (version 38.22) (65, 66). The fasta file was formatted as a single-line multiline fasta using fastx_toolkit (version 0.0.14), and CD-HIT (version 4.7) was used to cluster the nucleotide sequence at 99% (67). The output fasta file entries were renamed by accession number.

**(ii) *In silico* analysis.** Amplicons were created *in silico* using seqkit (version 1.4) amplicon. These targeted the VP1 capsid region for norovirus (64), resulting in 340/344-bp amplicons for norovirus GII and GI, respectively (see supplemental material for primer sequences). Some GIII, GIV, and GV sequences were generated using the specified primers. Adapter sequences were added at either end of the amplicons using seqkit (version 1.4) mutate, and seqkit (version 1.4) seq was used to filter amplicons to 400 to 500 bp in length (65).

For 40 samples, 10 simulated sequencing runs were designed, with various numbers of genotypes/sample. Reads/sample were set using the *R* package EnvStats (version 2.7.0), setting a truncated log-normal distribution, with a maximum of 5 million reads to be distributed across 40 samples. Input fasta files were compiled for each sample, and barcodes were added using seqkit (version 1.4) mutate.

**FIG 7:**
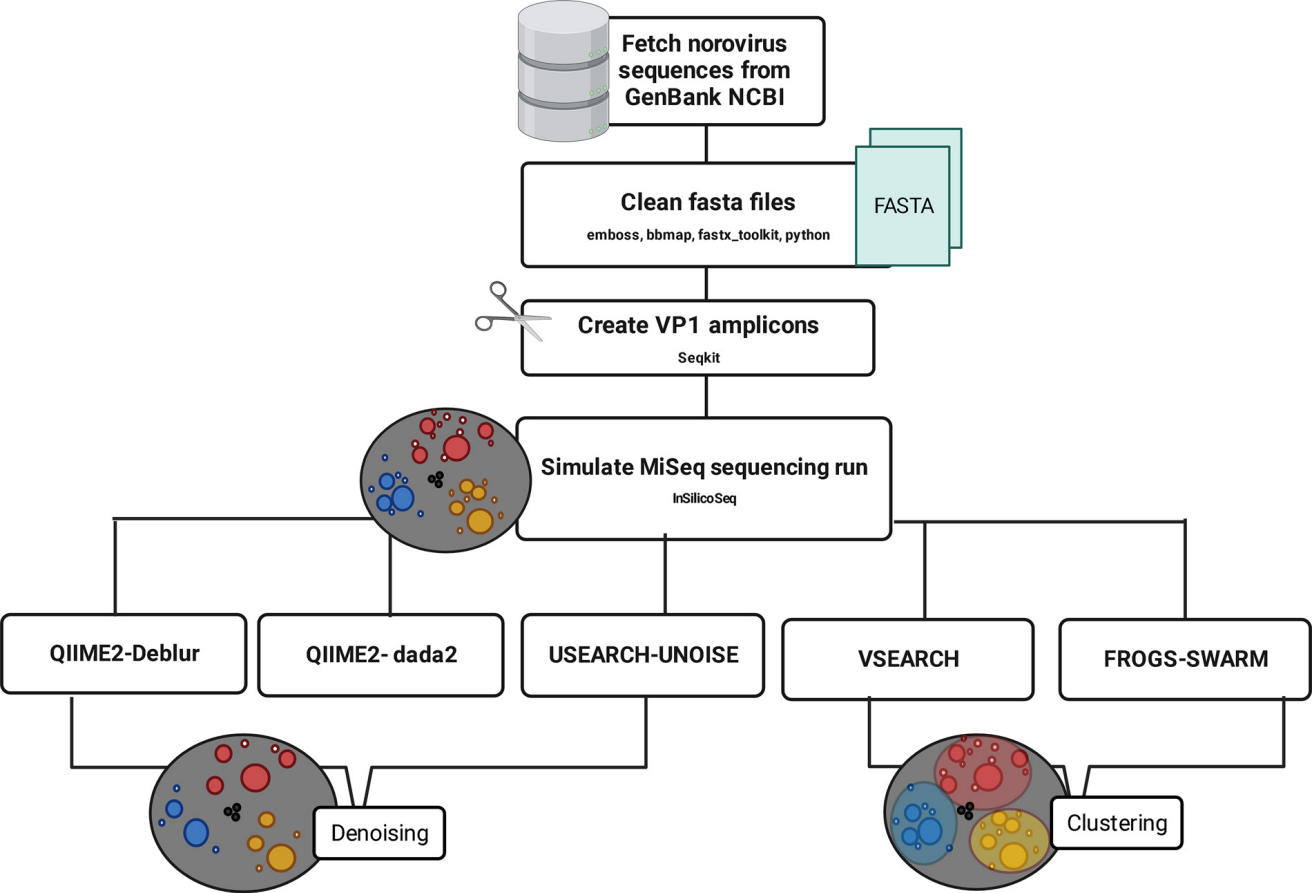
Schematic representation of the process used to generate amplicon sequencing data.

Simulations were performed using InSilicoSeq in python3.7 (66). A zero-inflated log-normal distribution was applied for reads assigned to each sequence in the input fasta file, on a paired-end 300-bp v3 MiSeq run. InSilicoSeq does not produce chimeric sequences. Sequences included in the simulations were classified externally using the NoroNet typing tool and included in the metadata file, alongside barcodes, accession ID, and the number of sequences/sample. Adapters and primers were trimmed from the simulation output using cutadapt (version 2.6) with an −*E* value of 0.2 and a minimum length of 100 bp. A schematic of the process is provided in Fig. 7.

**(iii) Bioinformatic analysis.** Trimmed fastq.gz files were imported into QIIME2 for DADA2 and Deblur-based analysis. For USEARCH-UNOISE3, VSEARCH and FROGS fastq.gz files were unzipped. Parameters set for the pipelines can be seen in Table 2.

**TABLE 2:** Parameters set for each pipeline

Parameters	Dada2	Deblur	FROGS	UNOISE3	VSEARCH
Phred quality score cutoff	20	20			
Trim: left	3				
Trim: right	5				
Minimum length		100		100	100
Maximum errors in forward reads	2				
Maximum errors in reverse reads	2				
Mean per nucleotide error		0.05			
Maximum mismatches alignment			20	20	20
Minimum abundance/sample		1	1	1	1
Minimum abundance, OTU/library			0		
Maximum length		260	344		400
Minimum overlap, bp		50	50	50	50
Clustering distance/similarity			1		99
Chimera removal	None	None	*De novo* UCHIME method	*De novo* UCHIME method	*De novo* UCHIME method and reference-based
Maximum expected error threshold				1	1

FROGS incorporates an affiliation step relying on a custom formatted RDP classifier. In this case, trimmed capsid sequences (VP1) amplicons were clustered using CD-HIT at 99% identity to reduce redundancy. The subsequent fasta file was classified to genotype and capsid designation, and entries without complete classification were removed. FROGS fasta2RDP.py was used to format the file as an RDP database. The fasta file was used to affiliate taxonomy for the clustered output, and taxonomy was assigned using both the RDP classifier and NoroNet typing tool.

For UniFrac analysis, files were imported into QIIME2/2021.2. Sequences were aligned using the MAFFT plugin and masked. For UniFrac analysis, rooted trees were generated using rooted fastree and distances computed with all tips.

Custom BLAST databases were created based on the expected data for each simulation. The observed output for each pipeline was blasted against the custom database, requiring 99% similarity at 75% coverage of the amplicon. Multiple hits for an observed sequence to a reference sequence in the BLAST db were filtered. The observed OTU with the highest bit score and the lowest *E* value on a per pipeline/simulation was selected for the comparison, if multiple hits were obtained. Blastout results were compared based on bit score using PERMANOVA and visualized using the *R* package ggheatmaps and complexheatmap.

### Classifier comparison.

**FIG 8:**
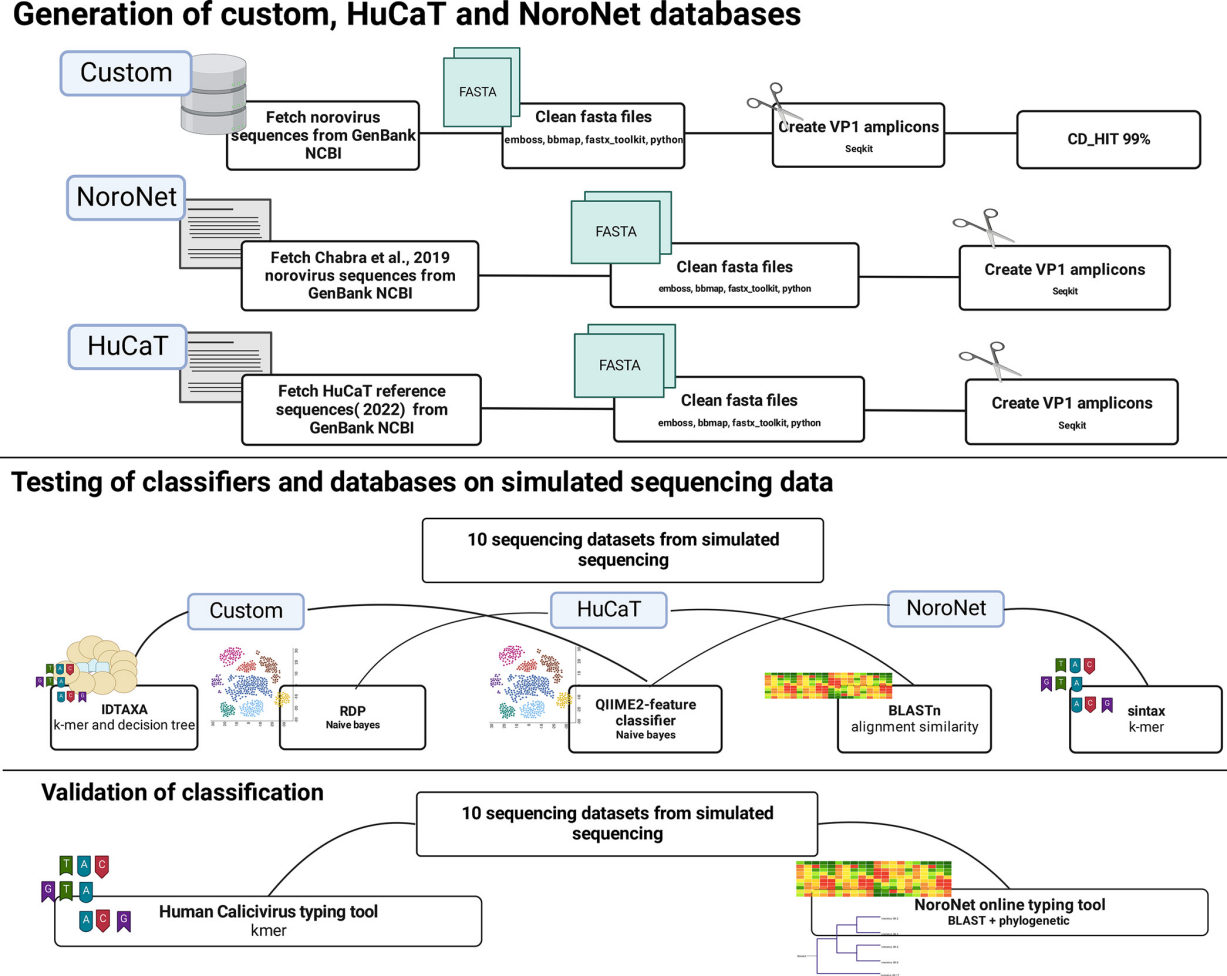
Schematic representation of the process used to compare classifiers and databases.

NoroNet and HuCaT databases were generated by fetching sequences from GenBank NCBI based on the provided accession numbers (2, 63) using the rentrez package in *R* (67). For the custom database, norovirus sequences available on GenBank NCBI but not included in the NoroNet/HuCaT database were clustered at 99% identity using CD-HIT (version 4.7). The resulting sequences were classified using the NoroNet typing tool, and no more than four sequences/genotype were kept in the final database (Fig. 8). Amplicons were created as per seqkit methods outlined in the previous section for all databases, and the amplicon databases were used to train the chosen classifiers.

The five classifiers chosen for comparison were IDTAXA, BLASTn, RDP, QIIME2 naive Bayes, and SINTAX. Confidence thresholds were set based on the classifier recommendations. IDTAXA (*R* package DECIPHER version 2.22.0) was run at 60% confidence threshold, while RDP (version 2.13) and SINTAX (usearch11 version 0.667) were run at 80% confidence threshold values. QIIME2 (version 021.2) naive Bayes were run at a 70% confidence threshold. BLASTn (24) uses various confidence thresholds for each taxonomic level, so confidence thresholds for species, family, and phylum were set to 90%, 70%, and 60%, respectively, with a percentage coverage of 50% of a reference sequence. Taxonomy assignment was validated against the NoroNet typing tool (62) and HuCaT typing tool (63).

A confusion matrix was generated using the yardstick package (version 1.0) in *R* from tidymodels (27, 68). A confusion matrix is a summary of prediction results on a classification problem. The number of correct and incorrect predictions are summarized with count values, by each class. The data were coded in a binary fashion, encoding 1 for agreement between expected and observed data and 0 for disagreement. Classifiers and databases were compared based on the sensitivity or true positive rate (TPR), false-positive rate (FPR), or 1-specificity, F1 score and balanced accuracy (average of sensitivity and specificity). Sensitivity refers to the probability of obtaining a positive test for a true positive and false positivity rate refers to the probability of obtaining a false-positive test for a true negative or in this case misclassification or missed classification. F1 score takes the harmonic mean of the sensitivity and specificity, while balanced accuracy takes the mean of the sensitivity and specificity. Jaccard distance measures (*R* package vegan version 2.6.2) were used to assess true and false matches between expected and observed data. Jaccard Similarity is a proximity measurement used to compute the similarity between two data sets, in this case, expected versus observed OTUs.

### Statistical analysis.

Distance matrices were conducted in *R* 4.1.3 using the *R* package vegan (version 2.6.2). All distance measures were conducted using 999 permutations (Bray-Curtis and Jaccard). Analysis of similarities (ANOSIM) and analysis of variance using distance matrices (ADONIS2) were also performed using the vegan package in *R* with Bonferroni *P*-value correction. Kruskal-Wallis and Wilcoxon tests were performed in base *R*, while the *post hoc* test for Kruskal-Wallis was conducted using the Dunn test in the *R* package rstatix (version 0.7.0). Post hoc tests for ANOSIM/ADONIS2 were performed using the *R* package RVAideMemoir (version 0.9–81-2).

### Data availability.

The scripts used for the simulation of sequencing data, processing of bioinformatic data, and classifier setup are available at https://github.com/ahfitzpa/Benchmarking-bioinformatics-norovirus-amplicons. The expected compositions and sequences for each simulation, as well as the classifier database inputs, are available at https://doi.org/10.5281/zenodo.7271067 for download.
