# Supplementary material for: Benchmarking Bioinformatic Tools for Amplicon-Based Sequencing of Norovirus
Source: Appl Environ Microbiol. 2022 Dec 21;89(1):e01522-22. doi: 10.1128/aem.01522-22 (PMC9888279; doi:10.1128/aem.01522-22)
Supplement: Supplemental file 1 — Supplemental material. Download aem.01522-22-s0001.pdf, PDF file, 1.2 MB [file aem.01522-22-s0001.pdf]

Supplementary material

Primers used for the generation of amplicons

GI Primers

GISKF: CTG CCC GAA TTY GTA AAT GA

GISKR: CCA ACC CAR CCA TTR TAC A

GII Primers

G2SKF: CNT GGG AGG GCG ATC GCAA

G2SKR: CCR CCN GCA TRH CCR TTR TAC AT

Supplementary Table 1. Kruskal-Wallis test results pertaining to the accuracy of genotypic assignment across expected relative abundance.

pipeline	n	statistic	df	p	method
dada2	153	94.4	19	5.37E-12	Kruskal-Wallis
deblur	168	109	19	1.22E-14	Kruskal-Wallis
FROGS	168	85	19	2.47E-10	Kruskal-Wallis
unoise3	169	102	19	2.18E-13	Kruskal-Wallis
vsearch	169	91	19	2.23E-11	Kruskal-Wallis

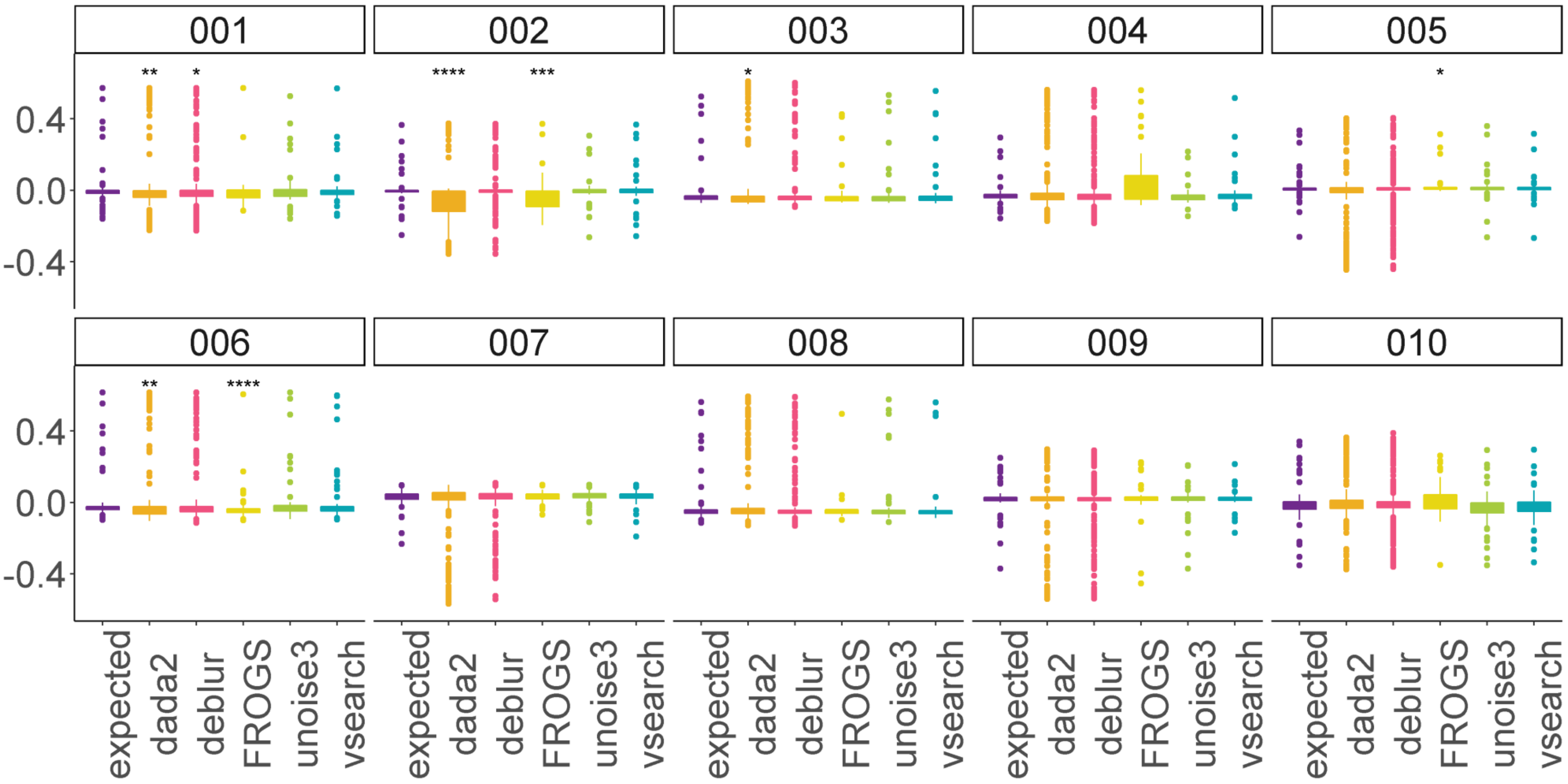

Supplementary Figure 1. Boxplots of Bray Curtis dissimilarity matrix based on the primary principle component illustrate the compositional dissimilarity demonstrate that DADA2 output was significantly different to the expected composition in 4/10 simulations, followed by Deblur and FROGS

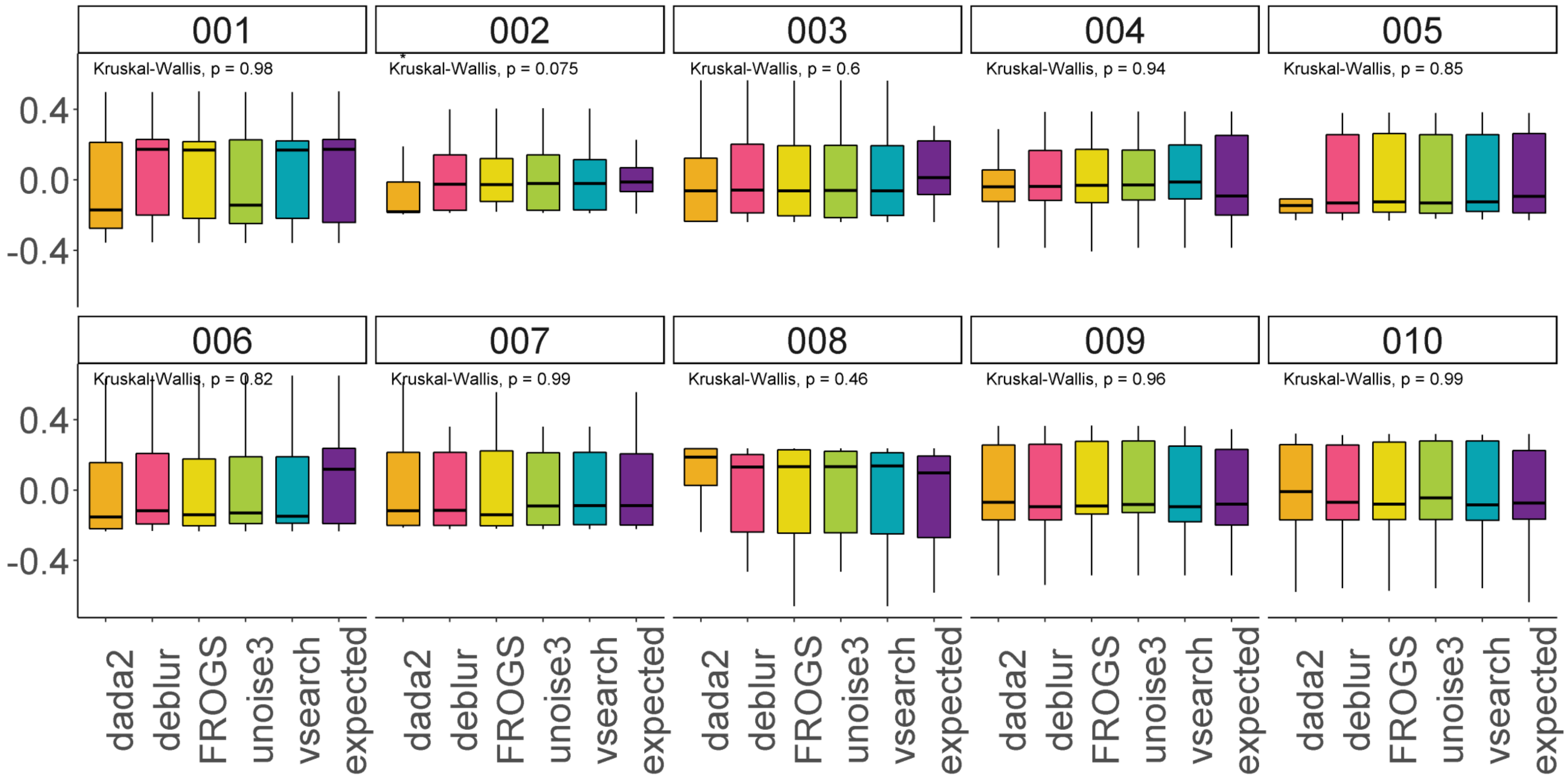

Supplementary Figure 2. Boxplots of unweighted UniFrac (QIIME2) based on the primary principle component illustrate the phylogenetic similarity between expected and observed sequences across all simulations

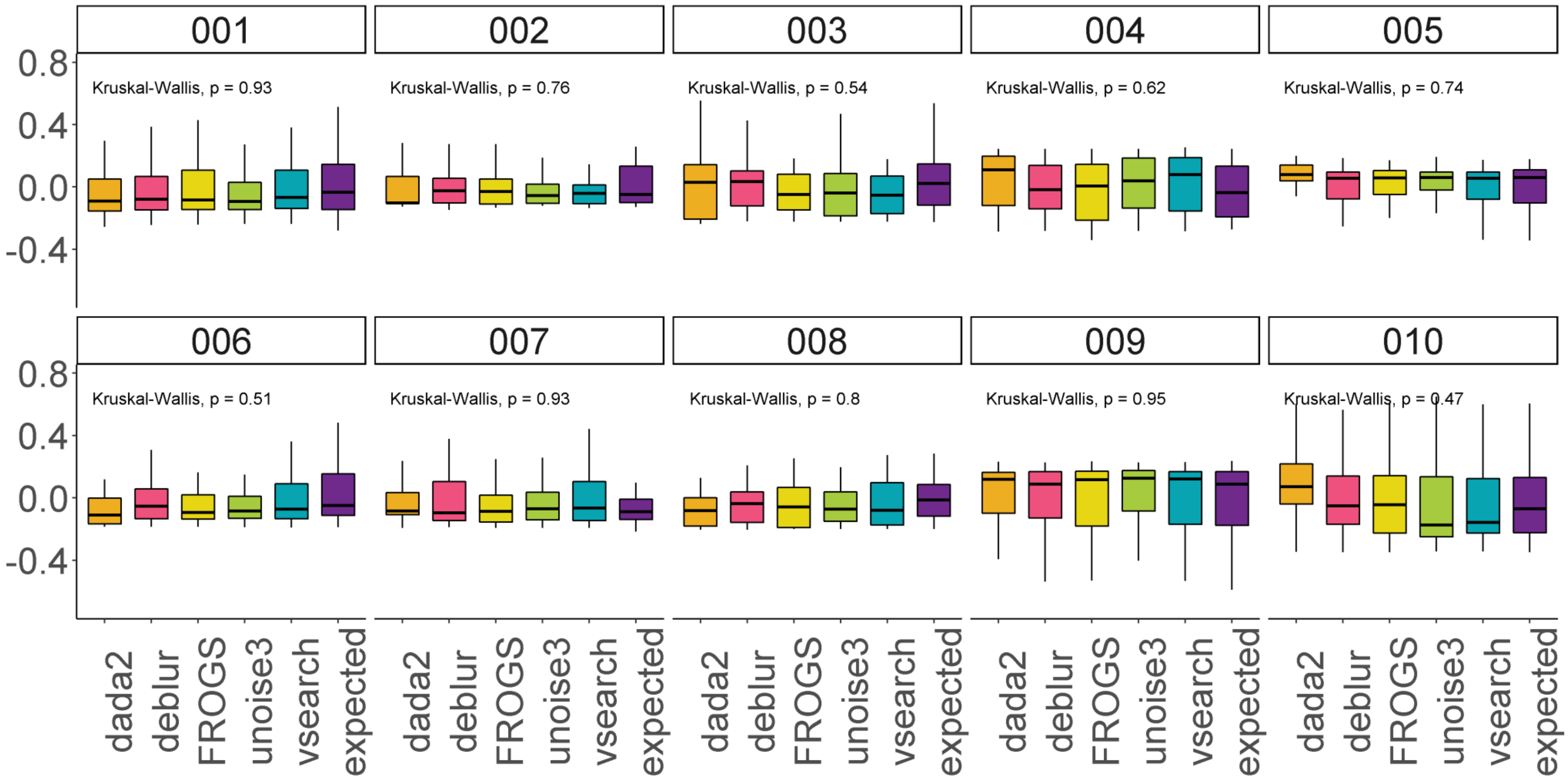

Supplementary Figure 3. Boxplots of weighted UniFrac (QIIME2) based on the primary principle component illustrate the phylogenetic similarity between expected and observed sequences across all simulations

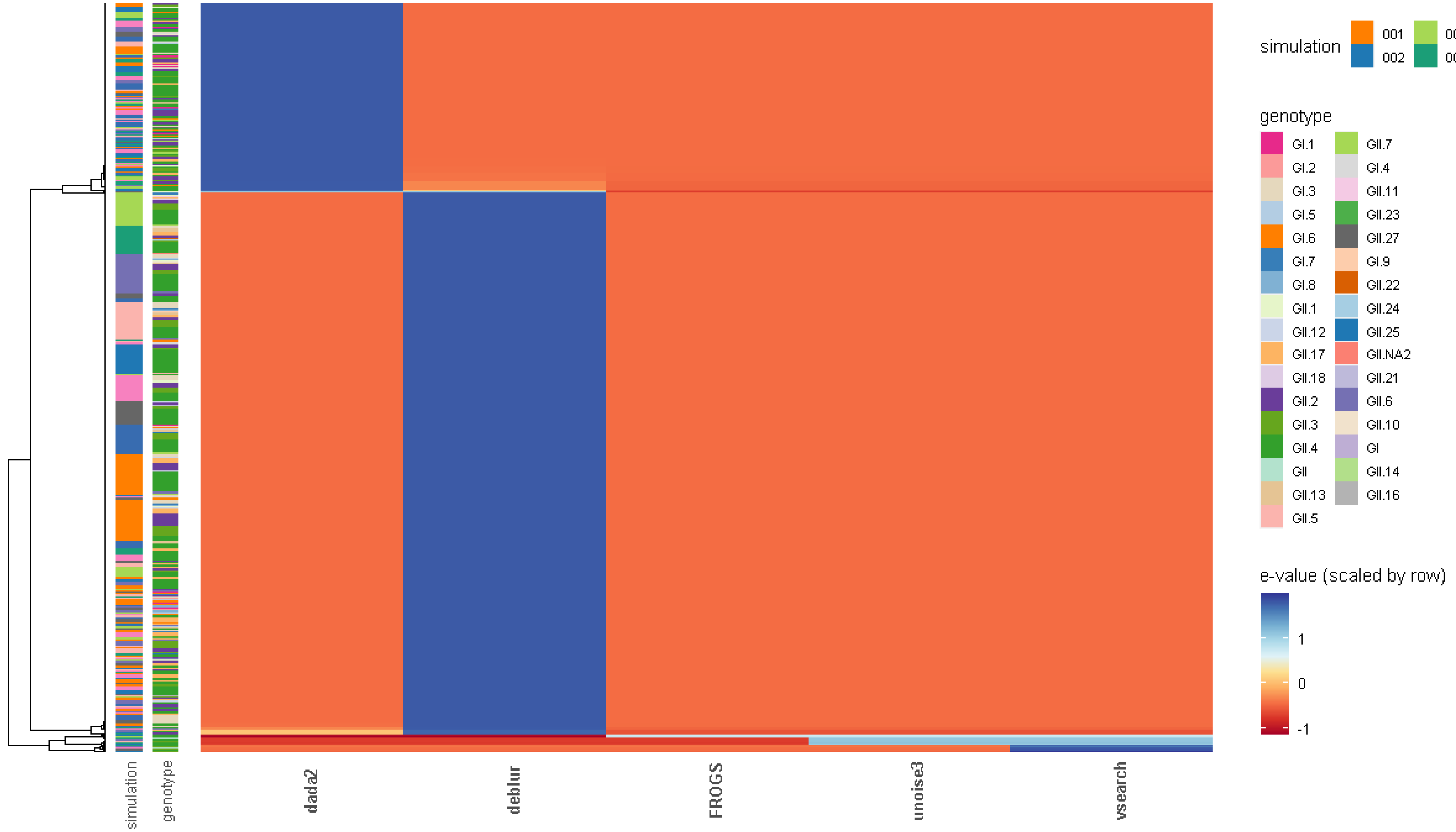

Supplementary Figure 4. Heatmap of e-value returned when observed sequences were subject to blastn at 99% identity with 75% coverage. E-values were scaled by row to clearly demonstrate differences.

Supplementary Table 2. Dunn post hoc test results for significant differently classifiers based on balanced accuracy

gold_standard	database	group1	group2	p.adj
Human Calicivirus Typing tool	custom	RDP	sintax	2.17E-06
NoroNet Typing Tool	custom	RDP	sintax	7.25E-06
NoroNet Typing Tool	HuCaT	IDTAXA	RDP	1.52E-05
NoroNet Typing Tool	NoroNet	IDTAXA	RDP	7.70E-05
Human Calicivirus Typing tool	HuCaT	IDTAXA	RDP	7.76E-05
Human Calicivirus Typing tool	NoroNet	IDTAXA	RDP	3.43E-04
NoroNet Typing Tool	HuCaT	blast	RDP	8.06E-04
Human Calicivirus Typing tool	HuCaT	blast	RDP	8.33E-04
NoroNet Typing Tool	HuCaT	IDTAXA	sintax	1.34E-03
NoroNet Typing Tool	NoroNet	blast	RDP	2.17E-03
Human Calicivirus Typing tool	NoroNet	blast	RDP	4.17E-03
NoroNet Typing Tool	NoroNet	IDTAXA	sintax	7.15E-03
NoroNet Typing Tool	custom	QIIME-NB	sintax	7.87E-03
NoroNet Typing Tool	custom	blast	RDP	8.16E-03
Human Calicivirus Typing tool	custom	QIIME-NB	sintax	9.03E-03
NoroNet Typing Tool	HuCaT	blast	sintax	3.15E-02
Human Calicivirus Typing tool	custom	blast	RDP	3.15E-02
Human Calicivirus Typing tool	HuCaT	IDTAXA	sintax	3.83E-02

Supplementary Table 3. Dunn post hoc test results for significant differently classifiers based on F1 score

gold_standard	database	group1	group2	p.adj
Human Calicivirus Typing tool	custom	RDP	sintax	8.26E-07
NoroNet Typing Tool	custom	RDP	sintax	1.29E-06
Human Calicivirus Typing tool	custom	RDP	sintax	2.17E-06
NoroNet Typing Tool	custom	RDP	sintax	7.25E-06
NoroNet Typing Tool	HuCaT	IDTAXA	RDP	1.52E-05
NoroNet Typing Tool	NoroNet	IDTAXA	RDP	7.70E-05
Human Calicivirus Typing tool	HuCaT	IDTAXA	RDP	7.76E-05
Human Calicivirus Typing tool	NoroNet	IDTAXA	RDP	3.43E-04
NoroNet Typing Tool	HuCaT	blast	RDP	8.06E-04
Human Calicivirus Typing tool	HuCaT	blast	RDP	8.33E-04
NoroNet Typing Tool	HuCaT	IDTAXA	sintax	1.34E-03
NoroNet Typing Tool	NoroNet	blast	RDP	2.17E-03
Human Calicivirus Typing tool	NoroNet	blast	RDP	4.17E-03
NoroNet Typing Tool	NoroNet	IDTAXA	sintax	7.15E-03
NoroNet Typing Tool	custom	QIIME-NB	sintax	7.87E-03
NoroNet Typing Tool	custom	blast	RDP	8.16E-03
Human Calicivirus Typing tool	custom	QIIME-NB	sintax	9.03E-03
NoroNet Typing Tool	HuCaT	blast	sintax	3.15E-02
Human Calicivirus Typing tool	custom	blast	RDP	3.15E-02
Human Calicivirus Typing tool	HuCaT	IDTAXA	sintax	3.83E-02

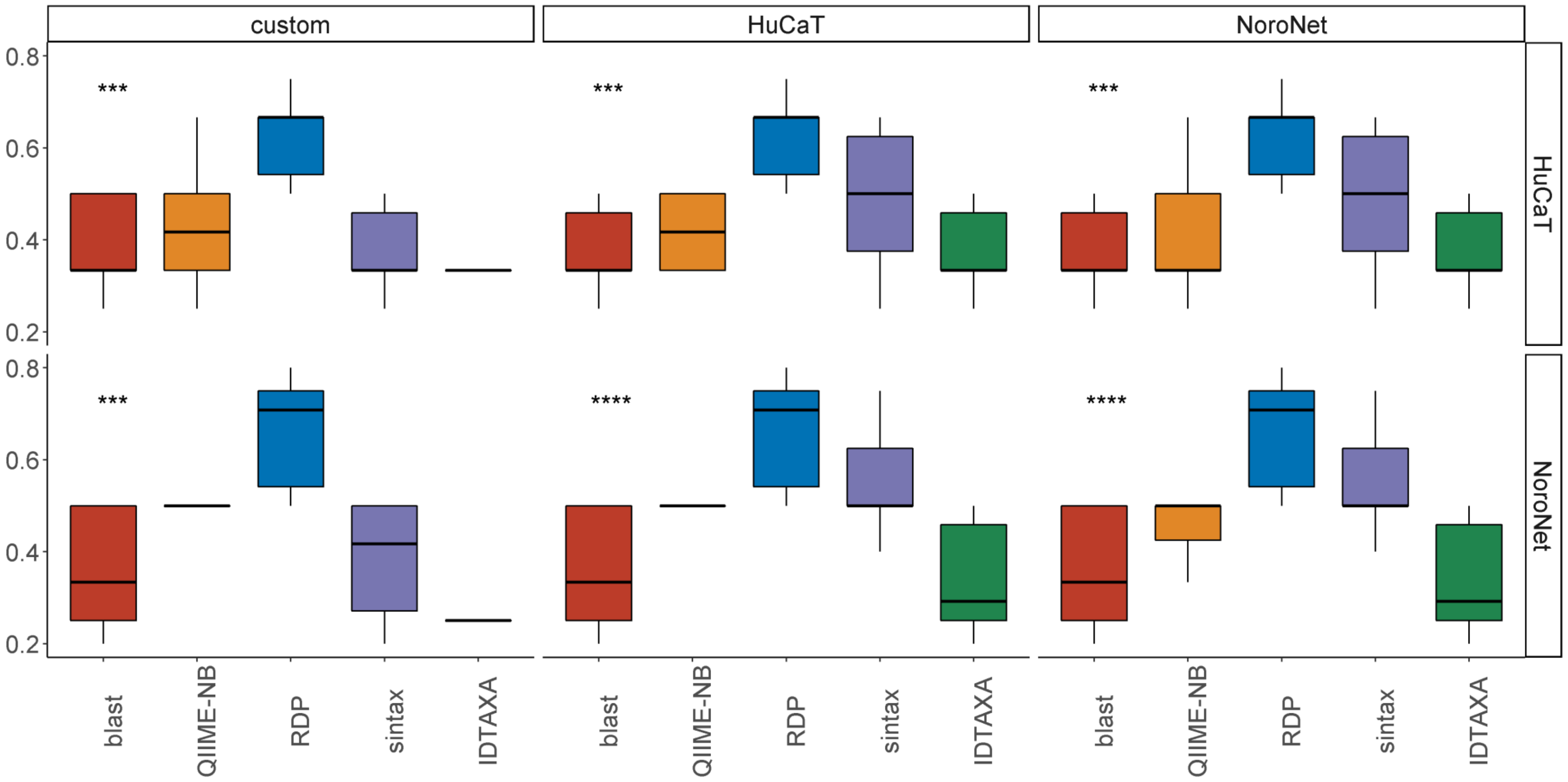

Supplementary Figure 5. Boxplot of True positive rate at taxonomic assignment to genotypic level. RDP performed well regardless of the underlying database, while the performance of sintax was strongly impacted by the choice of database, see the panel for custom vs NoroNet.

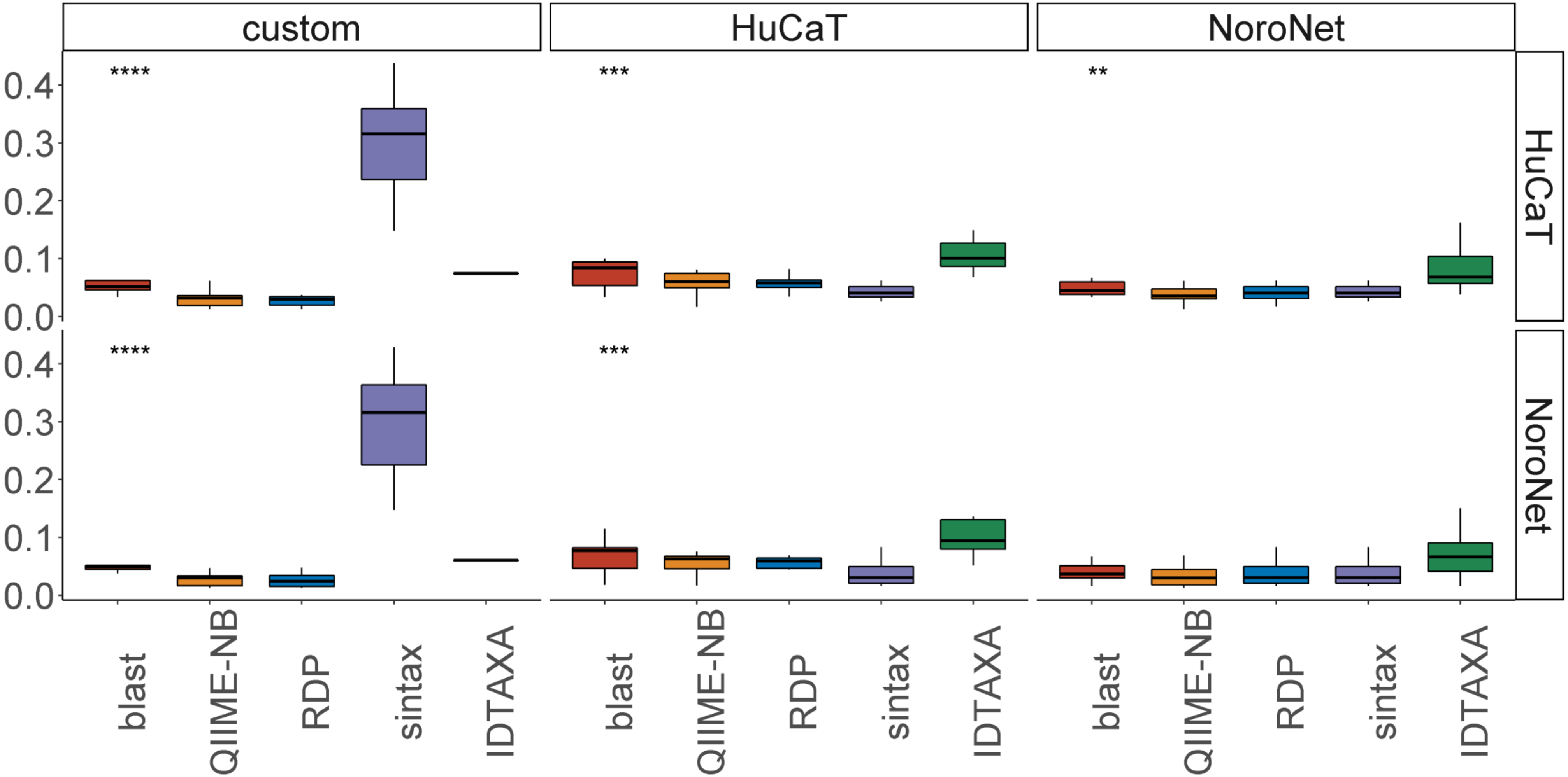

Supplementary Figure 6. Boxplot of false positive rate (1- specificity) at taxonomic assignment to genotypic level. QIIME2-RDP and RDP exhibited a similar performance across databases. Again, sintax performance was strongly impacted by the training database. There were no significant differences between the FPR of classifiers run using the HuCaT database.

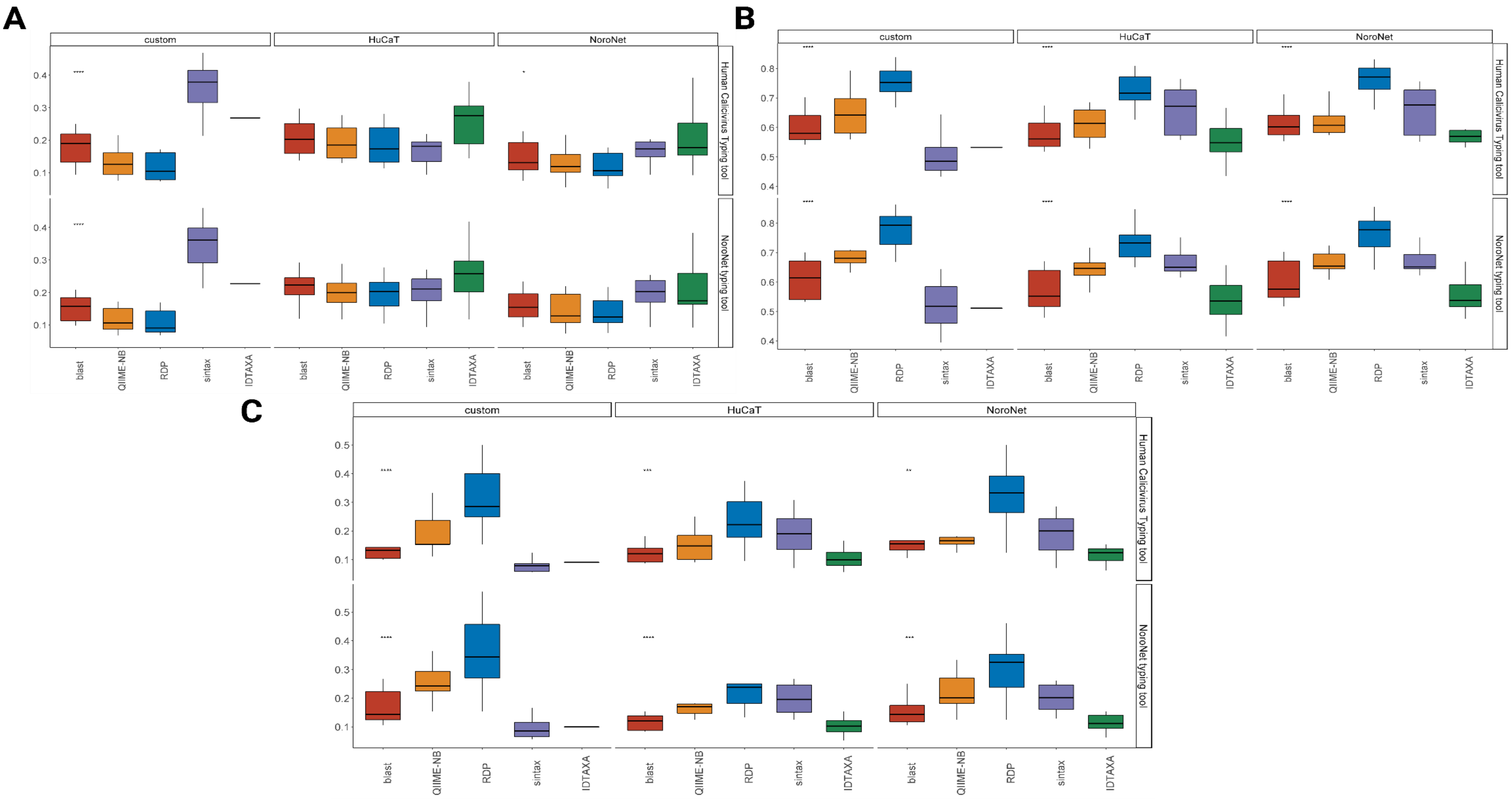

Figure 7. A. **Boxplot** of false positive rate (1- specificity) at taxonomic assignment to genotypic level. QIIME2-RDP and RDP exhibited a similar performance across databases. Again, sintax performance was strongly impacted by the training database. There were no sig differences between the FPR of classifiers run using the HuCaT database. **B. Boxplot** of balanced accuracy at taxonomic assignment to genotypic level. Classifiers were significantly different from one another (Kruskal-Wallis test). **C. Boxplo**t of F1 harmonic score at taxonomic assignment to genotypic level. Classifiers were significantly different from one another (Kruskal-Wallis test).
